# Integrated Deep Learning Surveillance of Unknown Pathogens with Pandemic Potential Using Pneumonia of Unknown Etiology

**DOI:** 10.3390/pathogens15040413

**Published:** 2026-04-10

**Authors:** Xiao Yang, Hui Ma, Min Zhu, Xinyu Song, Jiahao Feng

**Affiliations:** 1 The Fifth Clinical College, Anhui Medical University, Hefei 230032, China; 2345012472@stu.ahmu.edu.cn; 2 Graduate School, Medical School of Chinese PLA, Beijing 100853, China; zhumin8120@163.com (M.Z.); songxinyu615@163.com (X.S.); garmendia@163.com (J.F.)

**Keywords:** pneumonia of unknown etiology, integrated surveillance, emerging infectious diseases, pandemic potential, deep learning, early warning, chest imaging text, multi-pathogen surveillance, predictive analytics, disease outbreaks

## Abstract

Background: Pneumonia of unknown etiology (PUE), defined as pneumonia cases without an identified pathogen at the time of clinical presentation, represents a critical clinical warning signal for emerging infectious disease (EID) outbreaks with pandemic potential. Yet, conventional pathogen-centric surveillance systems suffer from an inherent blind spot: they cannot detect early clustering signals before the causative agent is identified, creating a window of vulnerability during novel pathogen emergence. To address this gap, this study aims to develop a deep learning model that leverages unstructured chest imaging text—a routinely available clinical data stream—to enable real-time, automated screening of PUE cases and early warning of EID clusters, independent of prior pathogen knowledge, within an integrated multi-pathogen surveillance framework. Methods: We retrospectively collected data from 8860 patients with respiratory illnesses at a tertiary hospital in Beijing, China, including 980 PUE cases (11.1%) and 7880 known-etiology pneumonia cases. A deep learning model (RoBERTa with attention enhancement) was developed using unstructured chest imaging reports. The Matthews correlation coefficient (MCC) curve was employed to determine the optimal decision threshold. Model performance was assessed for PUE case identification and clustering signal detection on a test set. Results: The model achieved an area under the receiver operating characteristic curve of 0.986 (95% CI: 0.981–0.991). At the optimal threshold of 0.08, selected by maximizing the Matthews correlation coefficient (MCC)—a balanced metric that accounts for all four confusion matrix outcomes—sensitivity was 89.8%, and specificity was 97.0% for identifying PUE cases. In a simulated surveillance exercise, the model showed a high correlation between the predicted and actual case counts (Pearson’s r = 0.901), suggesting its potential to detect abnormal clustering signals prior to pathogen identification. Conclusions: The developed model demonstrates potential to detect clustering signals of PUE caused by unknown pathogens and can be integrated with hospital information systems, providing a feasible, low-cost tool for integrated surveillance of pathogens with pandemic potential. This approach enables earlier outbreak detection and supports public health decision-making during the critical window before pathogen identification.

## 1. Introduction

Over the past two decades, emerging infectious diseases (EIDs), such as severe acute respiratory syndrome (SARS), Middle-East respiratory syndrome (MERS), and COVID-19, have continuously posed severe challenges to global public health security, causing significant health burdens and substantial socioeconomic impacts [1,2,3,4]. In the prevention and control framework for such diseases, robust surveillance systems capable of early warning and real-time monitoring are fundamental for interrupting transmission chains, reducing infection risks, and lowering mortality rates. However, achieving this goal is hindered by a critical challenge: in the absence of rapid and specific pathogen detection methods, cases for which the causative agent cannot be promptly identified are often classified as ‘pneumonia of unknown etiology (PUE)’ in the early stages of an epidemic. Consequently, PUE, as a clinical-epidemiological entity representing cases without an identified etiology at presentation, represents an important clinical warning signal for EID outbreaks. Syndrome-based surveillance systems, which monitor pre-diagnostic clinical indicators rather than laboratory-confirmed pathogens, have long been recognized as valuable tools for early outbreak detection in epidemiological practice [5,6].

The global COVID-19 pandemic further validated this observation. In the early phase, traditional pathogen-based surveillance systems failed to detect clustering signals due to insufficient testing capacity for the novel pathogen [7,8]. This led to delayed recognition and missed opportunities for early containment. Retrospective analyses have demonstrated that surveillance systems relying on confirmed case reports—the cornerstone of conventional infectious disease monitoring—are inherently ill-equipped to detect the emergence of novel pathogens. Such systems are designed for known diseases rather than for identifying anomalous signals from routine clinical data streams [9]. Collectively, these findings underscore a fundamental limitation of monitoring networks that depend solely on pathogen confirmation: their inherent lack of both timeliness and sensitivity to early signals.

In contrast, alternative data streams have shown promise for early outbreak detection. Studies leveraging syndromic surveillance approaches have demonstrated that electronic data sources, such as healthcare utilization records and internet search queries, can anticipate epidemic waves by up to two weeks before official case-based alerts [10,11]. Furthermore, data science methodologies applied to time-series case data have shown the feasibility of detecting early warning signals of disease re-emergence through statistical indicators such as variance and autocorrelation. These indicators can identify critical transitions in epidemic dynamics prior to exponential growth [11,12]. These findings highlight the urgent need for predictive analytics tools that can mine abnormal patterns in real time from routinely generated clinical data streams. Such tools must be capable of operating independently of prior pathogen knowledge, thereby addressing the unavoidable monitoring gaps created by conventional pathogen-centric surveillance models during the emergence of unknown pathogens.

Currently, pathogen-based surveillance primarily relies on highly specific detection techniques such as polymerase chain reaction (PCR), antigen testing, and metagenomic sequencing [13,14,15]. Although these methods are regarded as the “gold standard” for pathogen identification, their application in real-world surveillance suffers from inherent time lags—ranging from hours for PCR to days for metagenomic sequencing—limiting their capacity to provide timely early epidemic warning. Retrospective epidemiological studies have confirmed that abnormal clustering signals already existed in hospital information systems before the COVID-19 pandemic was officially recognized, yet traditional surveillance systems failed to capture them in a timely manner [16]. This highlights a fundamental limitation of monitoring networks that depend solely on pathogen confirmation: their inherent lack of both timeliness and sensitivity to early signals. Therefore, there is an urgent need for predictive analytics tools that do not rely entirely on pathogen identification, but instead can mine abnormal patterns in real time from routinely generated clinical data streams.

In recent years, with the rapid development of artificial intelligence, the use of machine learning and natural language processing (NLP) technologies to analyze electronic health records (EHRs) and medical text data to support clinical decision-making and public health surveillance has become an active research field [17,18,19]. Some studies have attempted to apply deep learning to the clinical diagnosis and management of COVID-19 [20,21,22,23,24]. Chen et al. established a deep learning-based diagnostic system for COVID-19 [25]. Wang et al. proposed COVID-Net for classifying chest X-rays as normal, pneumonia, and COVID-19 [26]. These studies demonstrated the potential of using EHR data for disease screening, highlighting its broader applicability for surveillance systems. For infectious disease surveillance specifically, NLP has been successfully applied to detect outbreak signals from electronic medical records and to identify symptomatic cases from physician notes in real time [27,28]. However, most studies on model optimization for surveillance systems still rely on traditional approaches that aim to balance accuracy or maximize the Youden index [29,30,31]. The default decision threshold (typically 0.5) employed in these conventional methods may not meet the high-sensitivity requirements essential for early warning of emerging infectious disease outbreaks. To achieve the overarching goal of minimizing missed detections in unknown pathogen surveillance—a critical objective within the public health surveillance context, where a single undetected cluster can seed a pandemic—there is an urgent need for model optimization strategies that prioritize high sensitivity.

This study identified the distinctive semantic features embedded in hospital chest imaging text reports, which are capable of differentiating between pneumonia of unknown etiology (PUE) cases and known etiology pneumonia (KEP) cases. (1) Model development and threshold optimization: By developing a deep learning model and employing a threshold optimization strategy that prioritizes high sensitivity for surveillance purposes, we aim to achieve real-time automated screening and early warning of PUE cases caused by unknown pathogens. Furthermore, in contrast to threshold optimization based on the F1 score [32] or selecting the optimal threshold via the precision–recall curve [33], we introduce the Matthews correlation coefficient (MCC)—a robust performance metric that incorporates all elements of the confusion matrix—to determine the optimal threshold for predictive analytics. The objective is to identify a decision threshold that ensures both high sensitivity and satisfactory overall predictive performance [34]. (2) Surveillance system integration and public health framework: In terms of application, this model can be integrated into hospital information systems as a backend service, automatically aligning with national surveillance rules for clustered PUE cases, thereby constructing an effective real-time surveillance and predictive analytics system. This study aims to elucidate the technical feasibility, performance evaluation, and integration pathways within the existing public health framework for achieving early intelligent surveillance of emerging infectious diseases caused by unknown pathogens based on clinical text records. The findings are intended to provide novel insights and empirical evidence to strengthen surveillance systems and predictive analytics capabilities for early warning of unknown pathogens, contributing to the development of a more intelligent infectious disease defense system.

## 2. Materials and Methods

### 2.1. Study Design and Data Source

This single-center retrospective observational study aimed to develop and validate a text model for real-time monitoring of PUE (Pneumonia of Unknown Etiology). The study subjects were recruited from a tertiary teaching hospital in Beijing, China, which serves as a regional referral center with several thousand inpatient beds. The hospital’s electronic health record system captures comprehensive clinical data, including radiology reports, laboratory results, and discharge diagnoses. We enrolled patients with a visit date between 1 January 2022 and 31 March 2023. Through the hospital’s electronic health record (EHR) system, we consecutively enrolled all respiratory disease patients aged 18 years and older who presented with respiratory symptoms, underwent chest imaging (including X-ray or CT), and had a confirmed final etiological diagnosis or clinical classification during the specified period. The exclusion criteria were: (1) severely missing or uninterpretable imaging report texts; (2) duplicate patient identity information. A total of 8860 patients were ultimately included in the study. Consecutive sampling was applied to minimize selection bias, ensuring that all eligible patients during the study period were included. The study period spanned multiple seasons (winter, spring, summer, autumn, and winter again), which may have introduced seasonal variations in respiratory pathogen distribution and disease severity; these factors are considered in the interpretation of the cohort characteristics.

For patients with multiple imaging examinations during the study period, only the single examination report closest in time to their etiological sampling (e.g., sputum culture, nucleic acid test) or final clinical diagnosis date was selected for analysis, as this minimizes temporal discordance between radiographic findings and the etiological reference standard, ensuring that the imaging features correspond most closely to the confirmed diagnosis. All imaging reports were stored in an unstructured free-text format, containing descriptive language covering the location, morphology (e.g., ground-glass opacity, consolidation, reticular pattern, fibrous streaks), density, distribution range, and other relevant imaging findings of lung lesions.

Based on the final etiological diagnosis or comprehensive clinical judgment, the collected cases were divided into two major categories:

(1) Known Etiology Pneumonia (KEP) Group: All pneumonia cases with pathogens identified by methods such as sputum culture, serological testing, and molecular nucleic acid testing (including multiplex PCR, targeted sequencing). This included bacterial pneumonia, other common and clearly identified viral pneumonias (excluding SARS-CoV-2), Mycoplasma pneumonia, Chlamydia pneumonia, etc.

(2) Pneumonia of Unknown Etiology (PUE) Group: The definition of PUE followed national relevant regulations: pneumonia cases meeting all four of the following criteria that cannot be clearly diagnosed as other diseases: (1) fever (axillary temperature ≥ 38 °C); (2) imaging features of pneumonia; (3) decreased or normal total white blood cell count, or decreased lymphocyte count in the early stage of illness; (4) no significant improvement or progressive worsening after 3–5 days of standardized antimicrobial therapy. To realistically simulate the early scenario of a new infectious disease outbreak, cases that were initially clinically diagnosed as “PUE” during the epidemic period due to delayed nucleic acid testing or unclear early etiology, but later confirmed as COVID-19 via SARS-CoV-2-specific nucleic acid testing, were included in this group. Consequently, the PUE group in this study comprised exclusively COVID-19 cases. This compositional choice introduces potential limitations regarding the generalizability of the model to PUE cases arising from other etiologies, which are addressed in Section 4.7.

To evaluate model training efficacy and generalizability, stratified random sampling was used to divide the complete dataset into three mutually exclusive subsets in a 7:1:2 ratio, with stratification performed based on the primary outcome (PUE vs. KEP) to preserve class distribution across subsets. The partitioning was performed as follows:

(1) Training Set (*n* = 6202): Used for learning and training model parameters.

(2) Internal Validation Set (*n* = 886): Used for hyperparameter tuning during training, monitoring model performance, and implementing early stopping strategies to prevent overfitting.

(3) Test Set (*n* = 1772): Completely unseen during model training and tuning, used solely for unbiased estimation of the model’s final performance.

The entire splitting process used a fixed random seed (seed = 42) to ensure reproducibility. No patient-level data from the test set were accessed at any point during model development or hyperparameter tuning. The dataset exhibited substantial class imbalance: overall, PUE cases comprised 11.1% (980/8860) and KEP cases 88.9% (7880/8860). Across the three subsets, the proportions of KEP and PUE were approximately consistent, with PUE accounting for about 11% in each subset. This balanced stratification preserved the original class proportion across subsets. To appropriately evaluate model performance under class imbalance, we report the precision–recall curve and average precision alongside conventional ROC-AUC.

### 2.2. Model Construction and Evaluation Metrics

This study constructed a deep learning text model. The model’s core task was to automatically classify whether an input chest imaging report text corresponded to a “PUE” or “KEP” case. The model architecture followed the design logic of “deep semantic feature extraction → key information enhancement → classification decision.”

#### 2.2.1. Text Feature Extraction Module

This module formed the foundation of the model, transforming unstructured text reports into numerical feature vectors rich in semantic information. We employed a RoBERTa model further pre-trained on Chinese medical text as the backbone network for feature extraction. RoBERTa is a pre-trained language model based on the Transformer architecture, possessing strong contextual semantic understanding capabilities. The input imaging report text was first preprocessed, including tokenization and conversion into input ID sequences and attention masks acceptable to the model. Subsequently, the text sequence was fed into the RoBERTa model, encoded through multi-layer Transformer encoders, outputting a hidden state sequence containing contextual information for each token. The hidden state vector corresponding to the special [CLS] token in this sequence was selected as the global semantic representation of the entire report. No additional projection layers were applied at this stage; the raw 768-dimensional [CLS] vector was passed directly to the attention enhancement module.

#### 2.2.2. Text Feature Attention Enhancement Module

To further enhance the model’s ability to focus on key information in imaging diagnostic text, an attention enhancement mechanism was introduced after the feature extraction module. Specifically, we applied an additive attention pooling layer to the full hidden state sequence output by RoBERTa [35]. Let $H=\left[h_{1},h_{2},\ldots ,h_{n}\right]$ denote the token-level hidden states output by RoBERTa, where n is the sequence length. The attention weight $\alpha_{i}$ for each token is calculated as

$$e_{i}=v^{⊤}tanh\left(W_{a}h_{i}+b_{a}\right),\;\alpha_{i}=\frac{\exp\left(e_{i}\right)}{\sum_{j=1}^{n}\exp\left(e_{j}\right)}$$

where $W_{a}$ and v are learnable parameters, and $b_{a}$ is a bias term. The weighted context vector is $c=\sum_{i=1}^{n}\alpha_{i}h_{i}$. The [*CLS*] vector is then extracted again from the sequence after applying these attention weights, yielding the final optimized text feature vector. This mechanism enhances descriptions highly relevant to PUE discrimination while weakening relatively redundant or irrelevant information.

#### 2.2.3. Classification Layer Design

The classification layer received the feature vector output by the attention enhancement module (which is the enhanced [CLS] representation of dimension 768) and performed the final binary classification task. This part consisted of a fully connected neural network: first, the feature vector underwent a non-linear transformation via a linear layer with output dimension 128, followed by a ReLU activation function. Subsequently, a Dropout layer (dropout rate set to 0.1) was connected to randomly discard some neuron connections as an effective regularization means to suppress model overfitting and enhance its generalization ability. Finally, a linear layer with an output dimension of 2 produced raw scores corresponding to the two categories: “PUE” and “KEP.”

#### 2.2.4. Model Training Process

The model was trained on a GPU-equipped server using the PyTorch (2022.1.3) deep learning framework and the Hugging Face Trainer API. Key hyperparameter settings were as follows: the AdamW optimizer was used with an initial learning rate of 5 × 10^−5^ and a weight decay coefficient of 0.01; the training batch size was set to 4 based on GPU memory; the total number of training epochs was set to 20, coupled with an early stopping strategy—training was terminated early if the loss on the internal validation set did not decrease for 10 consecutive epochs to avoid overfitting. The loss function used was cross-entropy loss. Text sequences were truncated to a maximum length of 256 tokens. A fixed random seed (42) was used for reproducibility [36]. The model weights that achieved the best validation loss were saved and used for final evaluation on the test set.

#### 2.2.5. Core Performance Evaluation Metrics

Centered on the clinical requirement of “prioritizing high sensitivity,” we constructed a multi-dimensional evaluation system. Core metrics included: (1) Sensitivity (Recall), the proportion of true PUE cases correctly identified by the model, which was the primary optimization goal of this study; (2) Specificity, the proportion of true KEP cases correctly excluded by the model; (3) Area Under the Receiver Operating Characteristic Curve (AUC), used to comprehensively evaluate the model’s overall discriminative ability across different decision thresholds; (4) F1 Score, the harmonic mean of precision and recall, used to balance the model’s overall performance; (5) Positive Predictive Value (PPV) and Negative Predictive Value (NPV), evaluating the model’s practical value from the perspectives of clinical review workload and screening reliability, respectively [37,38,39]. All performance metrics are reported with 95% confidence intervals, calculated using 2000 bootstrap resamples of the test set. Given the substantial class imbalance in our dataset (11.1% PUE), we additionally adopted the Matthews correlation coefficient (MCC) for threshold optimization, as it provides a balanced measure that accounts for all four confusion matrix outcomes and is robust to imbalanced class distributions.

### 2.3. Determination of Model Classification Threshold

The raw probabilities output by the model need to be converted into final classification results via a decision threshold. Threshold selection is key to balancing model sensitivity and specificity, particularly crucial for this study, aiming at high-sensitivity screening. To select the optimal decision threshold, we plotted the performance curve of the Matthews Correlation Coefficient (MCC) against the decision threshold on the validation set. Analysis showed that on the validation set, the model achieved the highest MCC value (0.821) when the decision threshold was 0.08. This threshold was therefore selected as the optimal classification cutoff. The final model performance was then evaluated on the test set using this fixed threshold of 0.08, without any further adjustment.

We thoroughly compared the differences in core screening metrics between the aforementioned threshold and the conventional default threshold in machine learning (0.5). Calculation results on the test set indicated that when the threshold was 0.08, the model’s recall (sensitivity) was 89.8%; whereas when using the conventional threshold of 0.5, the model’s recall was only 75.0%. This comparison shows that adjusting the threshold from 0.5 to 0.08 increased the model’s ability to capture true PUE cases by approximately 14.8 percentage points. Although lowering the threshold is usually accompanied by a moderate decrease in specificity, in public health surveillance scenarios, the cost of missing one true PUE case—potentially triggering community transmission—far outweighs the increased clinical review cost from one false alarm case. Therefore, this threshold adjustment is necessary in the context of early outbreak warning.

Based on the above analysis on the validation set, the threshold of 0.08 was ultimately determined as the classification decision threshold for model deployment because it achieved the best overall performance (highest MCC) while significantly increasing model sensitivity to a level meeting screening requirements (close to 90%). This selection process ensured that the threshold decision was based on objective performance evaluation while closely aligning with the clinical application goal of high-sensitivity screening. The core performance metrics subsequently described are all final results calculated on the independent test set based on this selected threshold (0.08), reflecting the model’s true generalization ability.

### 2.4. Model Practical Application and Validation

This study designed a scheme for integrating the model as a backend real-time service into the Hospital Information System (HIS), simulating real-world deployment and demonstrating the model’s utility in surveillance of emerging viral pneumonia. This scheme is deployed as a background process, continuously monitoring newly generated chest imaging reports in the hospital’s Radiology Information System (RIS) or Electronic Medical Record (EMR). After each new report is generated, the text is automatically extracted and input into the trained model for real-time analysis, with the model outputting its predicted probability of being PUE. According to the definition of “clustered PUE cases” in the National Surveillance, Investigation, and Management Plan for Pneumonia Cases of Unknown Etiology, the alert threshold is set at two or more epidemiologically linked PUE cases occurring within the same medical institution within a two-week period. This threshold—while intentionally low to maximize sensitivity for early outbreak detection—may generate frequent false alerts in real-world deployment. In practice, this rule is intended to serve as an initial screening trigger, with subsequent verification steps (e.g., manual epidemiological investigation) required before formal public health action.

The system maintains a rolling 14-day time window, continuously tracking cases predicted as PUE-positive by the model. For preliminary epidemiological linkage, the system applies a rule-based algorithm using structured data fields available in the electronic health record. Specifically, two cases are considered preliminarily linked if they meet both of the following criteria:

(1) Spatial proximity: The cases are associated with the same inpatient ward, outpatient clinic, or residential community (based on recorded address information), or their geographic coordinates fall within a predefined radius (e.g., 500 m) using geocoded residential addresses.

(2) Temporal proximity: The dates of hospital visits (or symptom onset, where available) are within 14 days of each other.

This rule-based linkage approach is intentionally designed to be highly sensitive, prioritizing early detection over specificity. Once the system detects ≥2 cases within the same medical institution within 14 days that are judged as PUE-positive by the model and meet the above preliminary linkage criteria, it automatically triggers a first-level warning signal. This signal is notified via an interface to the hospital’s infection control department and the local Center for Disease Control and Prevention, initiating further field epidemiological investigation, targeted etiological testing, and emergency response procedures. The low threshold and sensitive linkage algorithm reflect the system’s role as a “sentinel” rather than a definitive diagnostic tool; all alerts are intended to prompt human review rather than automated intervention.

### 2.5. Ethical Statement

This study was conducted in strict accordance with the principles of the Declaration of Helsinki and was approved by the Medical Ethics Committee of the Chinese PLA General Hospital (Approval No.: S2023-746-01). The research involved retrospective analysis of fully de-identified clinical data, conducting secondary analysis only on previously completely anonymized medical records. There was no possibility of re-identifying individuals, nor any foreseeable harm to participants’ rights, health, or privacy. The original data collection process had obtained corresponding informed consent and usage authorization as required. During the research process, all data underwent systematic anonymization before extraction and analysis, removing all personal identifiers, including patient name, ID number, hospitalization number, contact information, and medical record numbers, to ensure patient privacy was not compromised. The above operations were performed on internal network servers, and research results were presented in aggregated form to avoid any potential identity disclosure risks. Furthermore, this study did not contain any images or other Supplementary Materials that could identify individuals; if future research involves such identifiable materials, we will obtain explicit written consent from the individuals in advance and submit the consent documents as an appendix.

## 3. Results

### 3.1. Basic Characteristics of the Study Cohort

This study included 8860 patients with respiratory diseases who met the criteria, with 980 cases (11.1%) in the PUE group and 7880 in the KEP group. Table 1 presents the baseline characteristics of the study population. In terms of demographic features, patients in the PUE group were older than those in the KEP group (66.4 ± 22.5 years vs. 53.1 ± 19.7 years, *p* < 0.001), and there was no significant difference in gender composition (33.8% female vs. 32.0%, *p* = 0.232). Regarding radiological text features, the PUE group showed a significantly higher proportion of ground-glass opacity/high-density shadow (57.4% vs. 35.5%, *p* < 0.001) and consolidation/air bronchogram/fibrous streak (21.8% vs. 17.3%, *p* = 0.001); however, there was no significant difference in the presence of tree-in-bud sign/miliary nodules/large mass/cavity/pleural effusion between the two groups (19.7% vs. 20.4%, *p* = 0.633). Peripheral/subpleural distribution, multiple lesions, diffuse distribution, and lower lobe involvement were slightly higher in the PUE group (0.8% vs. 0.4%, *p* = 0.047). Laboratory tests revealed that inflammatory markers (CRP, IL-6, WBC) and the percentage of neutrophils were significantly elevated in the PUE group, while lymphocyte count and percentage were significantly decreased, and platelet count was higher. There were also multiple differences in red blood cell-related indicators between the two groups (see Table 1). The above baseline differences provided a basis for distinguishable clinical features for the subsequent deep learning model constructed based on radiological text, especially the significant distribution differences in radiological manifestations between the two groups, which directly supported the possibility of the model learning discriminative patterns from the text.

### 3.2. Optimal Threshold Determination Using the MCC Curve

**Figure 1:**
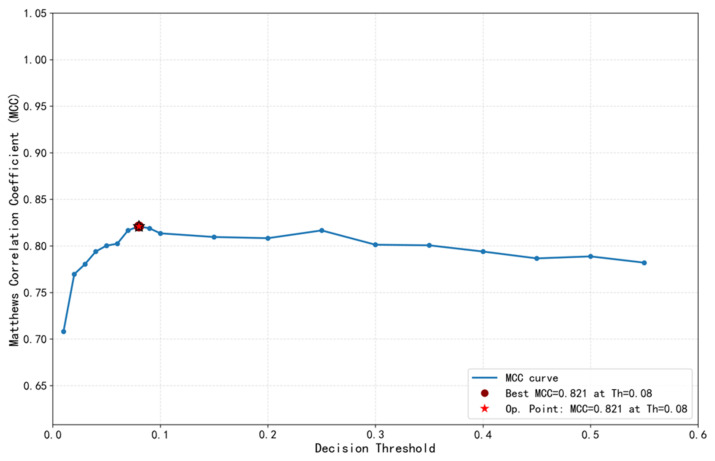
MCC Curve on the Validation Set for Threshold Selection. This figure illustrates the variation in the Matthews correlation coefficient (MCC) across different decision thresholds calculated on the validation set. The highest MCC value (0.821) is achieved at a threshold of 0.08, which was therefore selected as the optimal classification cutoff. The final model performance (sensitivity 89.8%, specificity 97.0%, etc.) was then evaluated on the independent test set using this fixed threshold. Compared with the conventional threshold of 0.5, this optimized threshold substantially improves sensitivity while maintaining high specificity.

**Figure 2:**
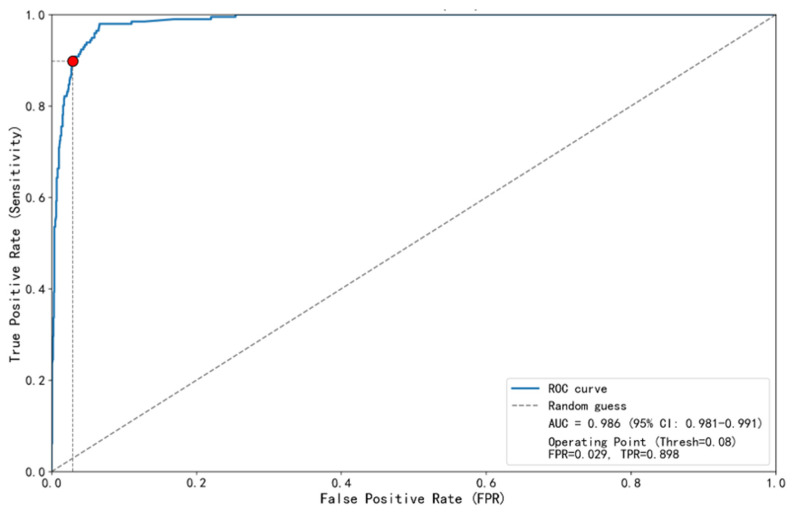
Area under the receiver operating characteristic curve for the pneumonia of unknown etiology screening model. This figure depicts the receiver operating characteristic curve of the model for discriminating between pneumonia of unknown etiology (PUE) and pneumonia caused by known pathogens (KEP) across all possible decision thresholds. The area under the curve was 0.986 (95% CI: 0.981–0.991), indicating excellent overall discriminative ability. The optimal decision threshold point (0.08), determined by maximizing the Matthews correlation coefficient, is marked on the curve. At this threshold, the model achieved a true positive rate (sensitivity) of 0.898 and a false positive rate of 0.029, corresponding to a specificity of 97.0%.

**Figure 3:**
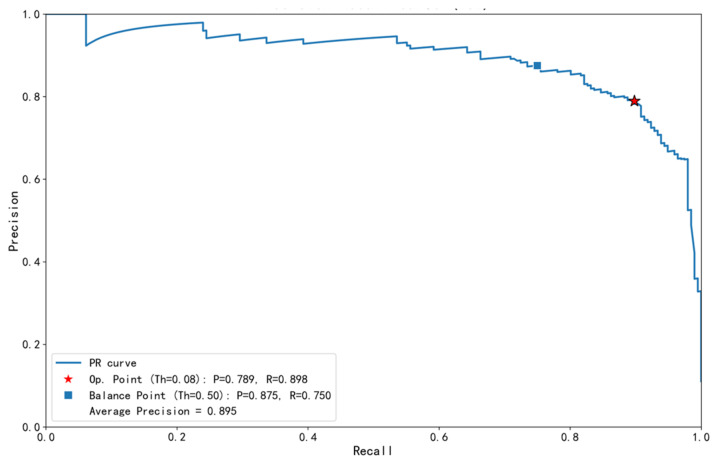
Precision–Recall curve of the pneumonia of unknown etiology screening model. This figure illustrates the trade-off between precision and recall across different decision thresholds, which is suitable for evaluating model performance in real-world screening scenarios where the distribution of the target class (pneumonia of unknown etiology, PUE) may be imbalanced. At the optimal decision threshold of 0.08, determined by maximizing the Matthews correlation coefficient, the model achieved a recall (sensitivity) of 0.898 and a precision of 0.789, meeting the core objective of this study to prioritize high sensitivity. In comparison, although the conventional threshold of 0.5 yields higher precision (0.875), its recall (0.750) is significantly lower, failing to meet the requirement for a low missed-detection rate in early warning systems. The average precision of 0.895 further confirms the model’s strong overall capability in identifying the target class.

To determine the optimal decision threshold for high-sensitivity screening, we evaluated the model’s performance across thresholds from 0.01 to 1.0 on the validation set (performance pattern analogous to that shown in Table 2). The Matthews correlation coefficient (MCC) curve on the validation set (Figure 1) showed a unimodal relationship, with the highest MCC value of 0.821 achieved at a threshold of 0.08. This threshold was therefore selected as optimal. The final model performance reported in this section (including Table 2 and Figure 2 and Figure 3) was then computed on the test set using this fixed threshold of 0.08, without any further threshold adjustment. Table 2 shows that at threshold 0.08, the model’s accuracy was 96.2%, sensitivity (recall) was 89.8%, and specificity was 97.0%. Compared to the conventional default threshold in machine learning (0.5), this optimized threshold significantly increased sensitivity by approximately 14.8 percentage points while maintaining high accuracy, with specificity only slightly decreasing from 98.7% to 97.0%. Table 2 indicates that at threshold 0.08, the model’s precision (PPV) was 78.9%, the negative predictive value (NPV) was 98.7%, and the F1 score reached 0.84, all representing excellent levels within the evaluated threshold range of this study. It is important to note that PPV is influenced by the prevalence of the positive class; in this test set, the prevalence of PUE was 11.1% (as reported in Section 3.1). Therefore, the observed PPV reflects performance under this specific class distribution and may vary in settings with different baseline PUE incidence. Compared to the conventional threshold of 0.5 (F1 = 0.808), the F1 score under the optimal threshold was higher, reflecting better overall performance.

The screening model developed in this study demonstrated strong discriminative ability for PUE on the test set. The Area Under the Receiver Operating Characteristic Curve (AUC) was 0.986 (95% CI: 0.981–0.991), indicating the model’s excellent discriminative ability (Figure 2). The Precision–Recall curve further assessed the trade-off in performance metrics under the high-sensitivity screening scenario (Figure 3). At the selected optimal threshold of 0.08, the model achieved a recall (sensitivity) of 0.898 and a precision of 0.789, with an Average Precision of 0.895, indicating the model’s good performance in identifying the target class. The threshold performance tables (Table 1 and Table 2) fully illustrate the trajectory of performance metrics with changing thresholds: as the threshold increased from 0.01 to 0.08, sensitivity gradually decreased from an extremely high 98.0% to 89.8%, while specificity steadily increased from 90.7% to 97.0%, and precision significantly improved from 56.8% to 78.9%. This dynamic process visually illustrates the trade-off between sensitivity and specificity through threshold adjustment.

### 3.3. Model Calibration and Risk Stratification

**Figure 4:**
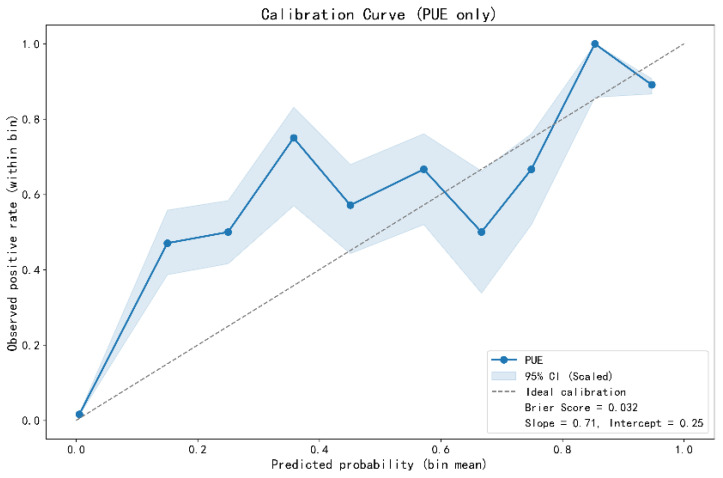
Calibration curve of the pneumonia of unknown etiology screening model (*n* = 1772). This figure evaluates the agreement between the model’s predicted probabilities for pneumonia of unknown etiology (PUE) cases and the observed positive rates through a calibration curve. The plot shows that the predicted probabilities closely align with the actual positive rates within each bin along the ideal calibration line. The model yields a Brier score of 0.032, indicating reasonable overall calibration. The calibration curve was constructed using fixed-width binning (10 bins) as implemented in sklearn.calibration.calibration_curve, where the average predicted probability and the observed positive rate are computed within each bin. However, the calibration slope of 0.71 suggests a tendency toward overconfidence in probability estimates, with predicted probabilities being too extreme relative to observed event rates. This pattern is consistent with a model optimized for high sensitivity, where probability estimates may be systematically shifted. In the context of a screening tool, this degree of miscalibration may be acceptable as long as the rank ordering of risk is preserved. A calibration slope of 0.71 reflects the model’s good discriminative ability in distinguishing cases with different levels of risk.

**Figure 5:**
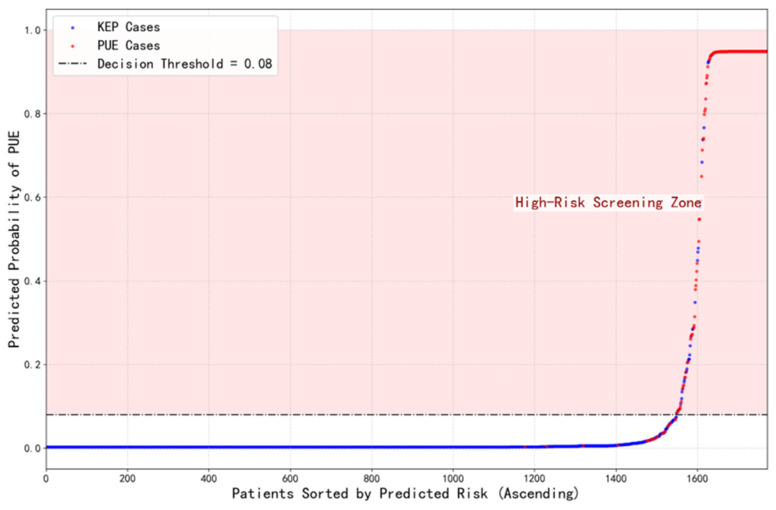
Distribution of predicted risk scores for PUE. This figure visually demonstrates the model’s risk stratification performance for the two categories—pneumonia caused by known pathogens (KEP) and pneumonia of unknown etiology (PUE)—by arranging all test cases in ascending order of their predicted risk scores. As shown, the predicted risk scores for the vast majority of KEP cases are concentrated in the lower range, particularly below the decision threshold of 0.08, whereas PUE cases are predominantly distributed in the higher-risk region above this threshold. This clear separation in distribution confirms that the features extracted by the model possess strong discriminatory power, enabling effective differentiation of the underlying risk between the two types of pneumonia.

The model demonstrated high reliability in clinical predictive risk stratification. The model calibration curve (Figure 4) showed a certain degree of consistency between the predicted probabilities and the observed true positive rate. The Brier score was 0.032, indicating the model’s fair discriminative ability. Further analysis of the fitting parameters, with a slope of 0.71 and an intercept of 0.25, suggested that the model overall tended to overestimate probabilities for high-risk cases and underestimate probabilities for low-risk cases. This is acceptable for a screening model that needs to capture potentially higher-risk cases as much as possible in the initial screening phase. The model’s reliability was further corroborated by the predictive risk distribution plot (Figure 5): as shown in Figure 5, the predicted risk values for the vast majority of KEP cases were concentrated below the decision threshold of 0.08, while PUE cases were mainly distributed above this threshold, showing that the model could perform clear and effective risk stratification of patients.

### 3.4. Model Clinical Utility and Screening Burden

**Figure 6:**
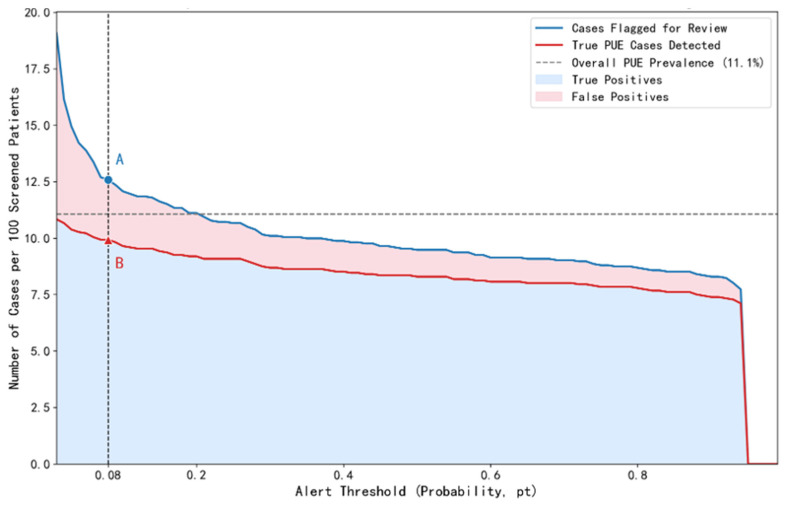
Clinical impact curve of the PUE screening model. This figure illustrates the dynamic trade-off between the clinical review workload (number of cases flagged for review) and the case detection benefit (number of true PUE cases identified) across different alert probability thresholds. As the classification threshold increases (moving rightward), the total number of cases requiring review (blue line) gradually decreases, while the number of true PUE cases detected (red line) also declines, indicating a progressive reduction in model sensitivity. The low threshold (0.08) selected in this study, although corresponding to a higher review burden, maximizes the capture of true PUE cases (true positive region). The false positive region (pink area) reflects the manageable cost of false alarms incurred to achieve high sensitivity.

**Figure 7:**
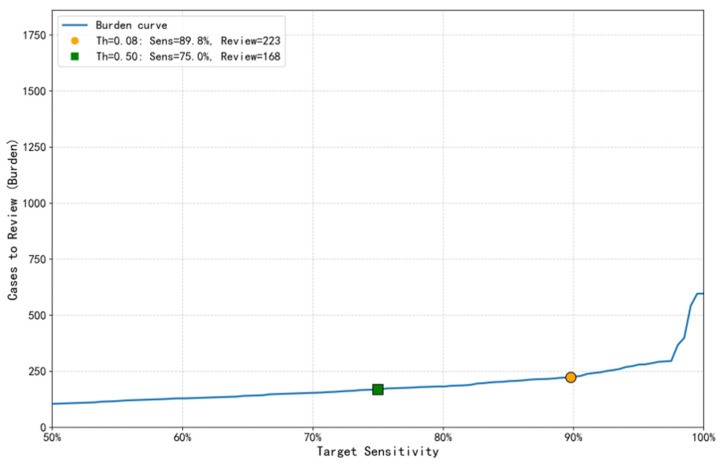
Screening burden versus detection capacity curve of the PUE screening model. This figure illustrates the increase in clinical review workload (burden) required by the screening model at different target sensitivity levels. When the conventional threshold of 0.5 is applied, the model achieves a sensitivity of 75.0%, requiring the review of 168 cases (9.5% of the test set). In contrast, using the optimized threshold of 0.08 in this study increases the sensitivity to 89.8%, with the number of cases for review rising to 223. This represents an additional review of only 55 cases (approximately 3% of the total cases), demonstrating that a significant gain in sensitivity can be achieved with a modest increase in workload.

To quantify the model’s potential impact in real clinical scenarios, this study systematically evaluated the trade-off between screening efficiency and review burden through the Clinical Impact Curve (Figure 6) and the Burden–Sensitivity Curve (Figure 7). The Clinical Impact Curve (Figure 6) shows that at the optimal threshold of 0.08, for every 100 imaging reports screened, the model would flag 12.6 high-risk cases for review, containing 9.9 true PUE cases and only 2.7 false positives, with a model sensitivity of 89.8%. This means clinicians only need to manually review about 12.6% of the reports to identify nearly 90% of the target cases, significantly improving screening efficiency. The Burden–Sensitivity Curve (Figure 7) further reveals that compared to reviewing 9.5% (168/1772) of total cases under the conventional threshold of 0.5, achieving close to 90% sensitivity with the optimized threshold of 0.08 required only an additional 3% review burden (55/1772). This indicates the model can achieve efficient case screening with a controllable clinical burden. This curve also clearly delineates the non-linear relationship between sensitivity improvement and review burden increase, providing an important basis for setting reasonable sensitivity targets in actual deployment. At the optimal threshold of 0.08, the false-alarm rate (the proportion of KEP cases incorrectly flagged as PUE) was 3.0% (1 − specificity), equivalent to 2.7 false positives per 100 reports screened (as shown in Figure 6). This level of false alarms is considered acceptable given the high sensitivity required for early outbreak detection.

### 3.5. Model Temporal Stability and Feature Space Visualization

**Figure 8:**
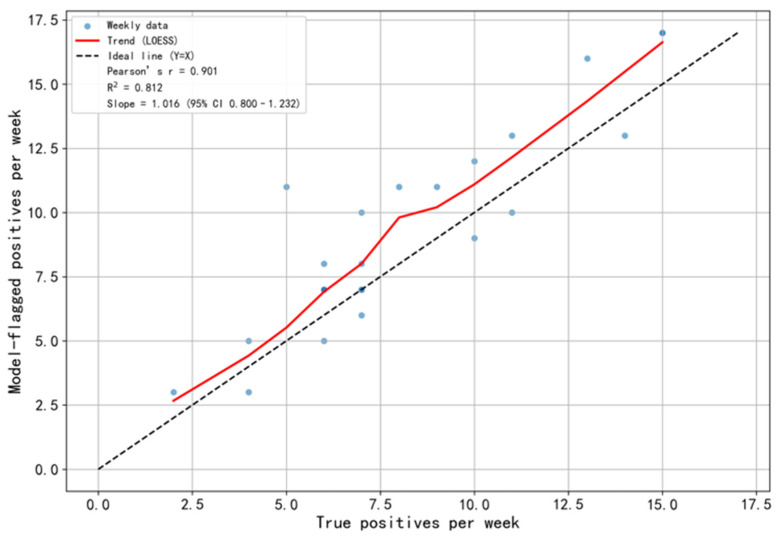
Simulated Weekly Surveillance Analysis. The figure shows a high positive correlation between the weekly number of predicted positive cases based on the optimal threshold (0.08) and the actual number of true PUE cases occurring each week. When the true case count is low (simulating “non-outbreak periods”), the model predictions fluctuate around a low baseline, primarily reflecting the false-positive level. When the true case count rises (simulating “outbreak periods”), the model predictions increase significantly, indicating that the increase is driven by true cases rather than random error. The scatter points cluster closely around the ideal Y = X reference line, demonstrating that the model maintains robust predictive consistency and low bias across different time periods.

**Figure 9:**
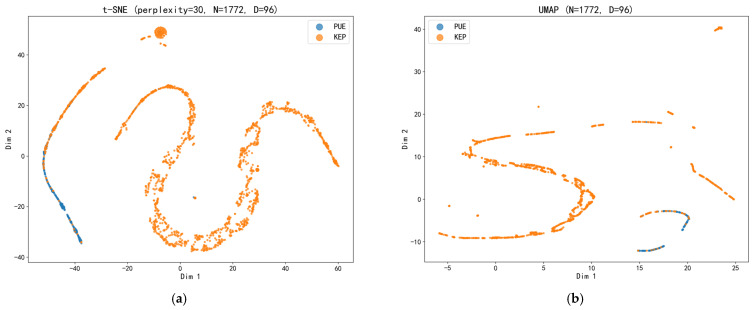
Visualization of the high-dimensional textual feature distributions of PUE and KEP based on (**a**) t-SNE and (**b**) UMAP dimensionality reduction. The left (**a**) and right (**b**) panels employ t-distributed stochastic neighbor embedding and uniform manifold approximation and projection, respectively, to reduce the high-dimensional semantic features extracted from chest imaging text reports into a two-dimensional plane, visually illustrating the distribution of PUE and KEP cases. Each point represents an individual patient, positioned by the two coordinates after dimensionality reduction, with color indicating the class label. In the low-dimensional embedding space, PUE and KEP cases exhibit a distribution pattern that shows both a trend of separation and partial overlap.

To assess the model’s long-term robustness and surveillance utility in a retrospective simulated deployment (rather than a prospective real-world implementation), this study conducted a time-series monitoring analysis using the historical data. The simulated weekly monitoring analysis plot (Figure 8) showed a high correlation between the weekly number of cases predicted by the model and the actual number of cases (Pearson’s r = 0.901, R^2^ = 0.812). The regression trend line slope (1.016) was close to the ideal reference line (Y = X), indicating that the model’s predictions were well-calibrated to the overall case volume over time. However, it is important to note that a strong correlation alone does not directly validate the model’s capacity to detect outbreaks in real time, as correlation does not account for the temporal ordering of predictions relative to actual events. Moreover, because this analysis was performed retrospectively on a fixed dataset, real-world deployment may introduce additional variability (e.g., changes in data distribution, reporting delays, or integration challenges) that could affect performance. The utility of such correlation should therefore be interpreted as supporting evidence of overall predictive consistency rather than as a definitive measure of early warning performance. No significant performance degradation or prediction drift was observed within the simulated time range of this study. These results preliminarily confirm the model’s potential as a tool for continuous monitoring of epidemic activity levels, with its alert frequency stably reflecting trends in community disease transmission risk. To further explore the intrinsic data basis of the model’s discriminative ability, this study used t-SNE (Figure 9) and UMAP methods (Figure 9) for dimensionality reduction visualization of the high-dimensional text features learned by the model. Visualization results showed that PUE and KEP cases exhibited a distribution pattern in the low-dimensional embedding space characterized by both separation and partial overlap. These visualizations are intended for qualitative illustration of feature distributions and should not be interpreted as quantitative evidence of model validity. The apparent separation in the embedding space is consistent with the model’s discriminative performance, but does not independently validate it.

## 4. Discussion

### 4.1. Main Findings

In the surveillance and early warning of emerging infectious diseases (EID) caused by unknown pathogens, the timely identification of clustered signals of pneumonia of unknown etiology (PUE) is critical for interrupting disease transmission. However, traditional surveillance frameworks that rely heavily on etiological confirmation are inherently limited by time lags that hinder real-time outbreak detection. This study aimed to overcome this limitation by evaluating the feasibility of a real-time early warning system based on clinical texts extracted from electronic medical records. Our findings demonstrate that the developed deep learning model exhibits robust discriminative capability in distinguishing between PUE and known pathogen pneumonia (KEP), achieving an area under the curve (AUC) of 0.986(95% CI: 0.981–0.991) on the test set. The Matthews correlation coefficient (MCC) curve identified 0.08 as the optimal decision threshold. At this threshold, the model achieved a sensitivity of 89.8% and a specificity of 97.0%, while both the F1-score (0.84) and MCC (0.821) reached their peak values. These findings demonstrate that the model achieves an optimal balance between overall classification performance and the high sensitivity essential for an effective screening tool within surveillance systems. Consequently, this study confirms the technical feasibility of leveraging unstructured radiology report texts for automated, high-precision screening and predictive analytics within epidemic surveillance systems. This approach offers a practical solution for enhancing early outbreak detection capabilities. Compared with previous AI-based surveillance systems that rely on structured data or ICD-10 codes, our model uniquely leverages unstructured chest imaging text to achieve early detection of PUE clusters without prior pathogen knowledge. While existing NLP-based systems have shown utility in detecting COVID-19 symptoms from physician notes or identifying outbreak signals from electronic medical records, the present study integrates a RoBERTa-based text encoder with an additive attention pooling layer and an MCC-optimized threshold tailored for public health surveillance [27,28]. The findings align with recent applications of machine learning in clinical risk prediction—such as models developed for the classification and diagnosis of chronic kidney disease [40]—further substantiating the reliability and translational potential of the methodology adopted in this research.

### 4.2. Baseline Characteristics and Their Implications for Model Performance and Surveillance Interpretability

Table 1 systematically compares the baseline differences in demographics, imaging features, and laboratory indicators between the PUE group (caused by unknown pathogens) and the KEP group. These characteristics provide essential context for understanding the surveillance population and the data foundation underlying the predictive analytics model. Regarding imaging characteristics, the PUE group exhibited significantly higher proportions of ground-glass opacity (GGO)/high-density opacity, consolidation/air bronchogram/fibrous strip opacities, and peripheral/subpleural distribution compared with the KEP group (all *p* < 0.05). These imaging differences constitute the fundamental data substrate for the subsequent deep learning model to effectively extract discriminative features from chest imaging reports. The model achieved an area under the curve (AUC) of 0.986(95% CI: 0.981–0.991) on the test set and maintained a sensitivity of 89.8% and a specificity of 97.0% at the optimized threshold of 0.08, objectively confirming that the discriminative information embedded in the imaging reports can be fully captured by the model. Nevertheless, deep learning models may rely on complex, non-intuitive latent feature combinations rather than directly mapping to specific radiological patterns; therefore, caution should be exercised when attributing model performance solely to individual imaging features, such as ground-glass opacity or consolidation. From a surveillance systems perspective, this high sensitivity at an early stage—before pathogen identification—enables the model to function as an effective pre-diagnostic screening tool for emerging infectious disease surveillance.

The imaging characteristics of the PUE group in this study are highly consistent with previous imaging studies on viral pneumonia. Existing research has confirmed that ground-glass opacities, peripheral distribution, and consolidations are common CT manifestations of COVID-19 pneumonia, whereas bacterial pneumonia more often presents as tree-in-bud signs and lobar consolidations. In this study, the PUE group primarily consisted of COVID-19 cases, and their imaging patterns were consistent with those previously documented. The KEP group comprised multiple etiologies, including bacteria, atypical pathogens, and non-SARS-CoV-2 viruses, resulting in more complex and heterogeneous imaging manifestations, yet still exhibiting distinct distribution patterns overall. This heterogeneity reflects the real-world complexity faced by epidemic surveillance systems, where multiple pathogen types circulate simultaneously and may present with overlapping clinical features.

Notably, some imaging signs previously considered highly associated with bacterial pneumonia (such as the tree-in-bud sign, cavity, and pleural effusion) showed no significant differences between the two groups in this study (*p* = 0.633). This phenomenon may be attributable to the case composition of the KEP group, which included a considerable proportion of non-bacterial pneumonia cases (such as mycoplasma, chlamydia, and other viral pneumonia), and these pathogens may not present the typical imaging characteristics classically associated with bacterial infection. This finding carries important implications for surveillance and predictive analytics: it indicates that relying on isolated single imaging features may be insufficient for accurate case classification in real-world epidemiological settings. Furthermore, it demonstrates that the model is operating in a real clinical scenario characterized by high etiological heterogeneity and partial overlap in imaging manifestations. That it achieves high discriminative accuracy under these challenging conditions further underscores the advantage of deep learning-based text modeling over traditional single-signature interpretation. This capacity to maintain robust performance despite real-world heterogeneity underscores the model’s potential as a reliable component of predictive analytics pipelines for epidemic surveillance.

The baseline analysis presented in Table 1 not only describes the basic composition of the study population but also provides intrinsic evidence for the validity of the model from the data source perspective. It simultaneously reveals the possible sources of the model’s performance boundaries—namely, the overlap between the two groups in certain imaging signs. This finding is consistent with the “separation with overlap” distribution pattern observed in the subsequent feature space visualization (Figure 9), enhancing the interpretability of the model’s decision basis. From a surveillance and predictive analytics standpoint, understanding these performance boundaries is essential for appropriate integration into public health workflows—the model functions as a highly sensitive “sentinel” for early warning rather than a definitive diagnostic tool, and its outputs should trigger further investigation rather than replace clinical judgment. This nuanced understanding of model capabilities and limitations is critical for the responsible deployment of AI-based surveillance tools in infectious disease epidemiology.

### 4.3. Determination of the Optimal Threshold for the Model

In integrating machine learning with healthcare systems for epidemic surveillance, threshold selection often directly determines the clinical utility and operational viability of the predictive model. Conventional practices frequently employ the default threshold (0.5) or the threshold maximizing Youden’s index, but these may not satisfy the high sensitivity requirements essential for infectious disease early warning systems, where missed detections carry substantial public health consequences. This study introduced the Matthews correlation coefficient (MCC) as a comprehensive performance metric to determine the optimal threshold, finding that MCC peaked (0.821) at a threshold of 0.08. In-depth analysis of the threshold-performance tables (Table 1 and Table 2) reveals that from 0.01 to 0.08, sensitivity decreased from 98.0% to 89.8%, while specificity increased from 90.7% to 97.0%, and precision substantially improved from 56.8% to 78.9%. This trajectory illuminates a critical trade-off in surveillance system design: a moderate reduction in sensitivity can yield substantial gains in specificity and positive predictive value—directly enhancing the credibility of warning signals and reducing unnecessary public health responses. Selecting 0.08 over a threshold yielding higher sensitivity (e.g., 0.01) reflects the operational logic of real-world surveillance: an effective early warning system must balance “detection capability” with “response feasibility.” An excessively low threshold would generate excessive false alarms, drowning authentic outbreak signals and exhausting limited public health resources for investigation and verification. The Burden–Sensitivity curve quantitatively confirmed that shifting from threshold 0.5 (sensitivity 75.0%) to 0.08 (sensitivity 89.8%) increased the review burden by only approximately 3%, demonstrating favorable cost-effectiveness for surveillance operations. Conversely, pursuing sensitivity above 95% would cause the review burden to escalate non-linearly and sharply, potentially overwhelming response capacity. Thus, 0.08 represents a “practical optimum” derived from empirical data—a threshold that appropriately balances epidemiological risk detection against the operational constraints of public health surveillance systems. This selection aligns with decision-theoretic principles for surveillance, where the operating point is chosen to minimize expected loss given asymmetric costs of false positives and false negatives, and is consistent with operational frameworks that prioritize sensitivity for rare but high-consequence events [41].

### 4.4. Application of the Model in Hospital Real-Time Surveillance Systems

The core value of surveillance systems and predictive analytics for emerging infectious diseases (EIDs) lies in their capacity to enable timely and effective public health interventions that control outbreak spread. In this study, we not only developed a high-performance predictive model but also designed a comprehensive surveillance framework aimed at automating and closing the loop for early warning of EIDs caused by unknown pathogens. The model is implemented as a backend service integrated into the Hospital Information System (HIS), enabling real-time analysis of newly generated chest imaging reports. This integrated design circumvents the delays associated with traditional manual reporting or data export. Furthermore, the early warning logic of this system references the national plan for the surveillance, investigation, and management of pneumonia of unknown etiology (PUE). The system continuously tracks a rolling 14-day time window and automatically triggers a Level 1 alert when the number of PUE-positive cases predicted by the model reaches or exceeds two, and preliminary epidemiological links can be established through electronic medical record data (e.g., personal details, ward location, consultation time). This automated process converts the textual directive—“≥2 epidemiologically linked PUE cases within 14 days”—found in the national plan into an executable computational rule. The significance of this technical implementation lies in its transformation of PUE surveillance from a “non-mandatory” task reliant on clinician memory and manual reporting into a “mandatory” active surveillance function that operates automatically and implicitly in the background of the information system, thereby strengthening predictive capacity for outbreak detection.

Nevertheless, several practical deployment challenges warrant consideration. First, computational latency: although the RoBERTa-based model achieves rapid inference (milliseconds per report) on GPU hardware, real-time processing of high-volume imaging streams may require optimization, such as batch inference or model distillation, to meet hospital throughput demands. Second, interoperability: seamless integration with heterogeneous HIS and radiology information systems (RIS) requires adherence to standards like HL7/FHIR and careful handling of varying data formats, which may necessitate middleware adaptation. Third, clinician acceptance: automated alerts risk contributing to alarm fatigue if not calibrated to local workflows; thus, user-centered design, transparent model explanations, and active engagement with infection control teams are essential to build trust and ensure appropriate responses. These issues should be addressed in future implementation studies.

### 4.5. The Role of the Model in the Early Warning System

A robust early warning and surveillance system for emerging infectious diseases should be multi-layered. The core value of our model and its integrated framework lies in filling the surveillance and predictive analytics gap that exists prior to definitive pathogen diagnosis—a function that can be conceptualized as the “broad-spectrum sensing layer” of an epidemic surveillance network. Its operational mechanism involves deep learning of pattern features from extensive chest imaging texts to enable real-time detection of spatiotemporal clusters of pneumonia of unknown etiology (PUE). These detected signals are then automatically aligned with national early warning criteria through the system’s built-in logic. Simulated surveillance analysis (Figure 8) provided supportive evidence for this mechanism: the number of cases predicted by the model showed a strong correlation with the actual number of cases (Pearson correlation coefficient = 0.901, R^2^ = 0.812). While this correlation suggests that the model’s predictions align well with overall epidemic trends, it does not, by itself, establish that the system can reliably generate early warning signals in real-world outbreak settings. The temporal dynamics of outbreak detection—specifically, whether predictions precede actual case increases—require further evaluation using time-lagged analyses or prospective validation. Nevertheless, these findings are consistent with the hypothesis that the system could contribute to early signal generation during the critical “blind window” when a novel pathogen first emerges, and specific diagnostic tests are not yet available. This approach—broad-spectrum screening and automated reporting based on clinical manifestations, followed by precise epidemiological investigations and laboratory testing by public health authorities—forms a complete closed loop for outbreak response. A similar prescreening concept has long been applied in influenza surveillance, such as using influenza-like illness (ILI) data to warn of influenza activity. Our study extends that concept to more complex, unstructured textual data and achieves full-process automation from screening to early warning trigger, thereby strengthening the predictive analytics capacity of infectious disease surveillance systems.

To prospectively evaluate this system in a real surveillance setting, we propose a multi-center prospective cohort study. The system would be deployed as a silent background monitor alongside existing surveillance workflows. Key evaluation metrics would include: (i) detection lead time compared to conventional pathogen-based reporting; (ii) prospective sensitivity and positive predictive value for identifying true PUE clusters; (iii) false alert rate per unit time; and (iv) clinician workload (e.g., alerts requiring manual review). The study would follow a stepped-wedge design, initially deploying in 2–3 hospitals with phased expansion. Statistical process control or prospective space-time scan statistics could be used to validate alerts. Such an evaluation would provide the necessary evidence for operational adoption.

### 4.6. Mapping Model Interpretability to Clinical Diagnostic Complexity

Deep learning models are sometimes criticized as invisible “black boxes,” hindering their clinical application in surveillance systems. This study provided a window into the model’s decision-making by visualizing high-dimensional features using t-SNE and UMAP (Figure 9). The visualization shows a distribution pattern of PUE and KEP cases in the feature space characterized by “separation with overlap.” This distribution pattern has profound implications for epidemic surveillance: the clear separation trend indicates that the model indeed learned combinations of textual features that can differentiate etiological patterns, with certain specific morphological description combinations more frequently associated with PUE cases requiring early warning. The partial overlap objectively reflects the inherent similarity in imaging manifestations among pneumonias of different etiologies, which is precisely the challenge in syndrome-based surveillance—balancing sensitivity against specificity. Importantly, this visualization not only enhances confidence in the model’s discriminatory capacity as a surveillance tool but also truthfully reflects its performance boundaries—it serves as a highly sensitive “early warning sentinel” within a surveillance system, not the definitive diagnostic “judge.” This aligns with the current trend in predictive analytics for epidemic surveillance, which emphasizes interpretability to facilitate public health decision-making. For example, Antoñanzas et al. also used feature importance analysis and local explanation methods when developing clinical prediction models to enhance trust in model-based surveillance outputs [42]. Our visualization results indicate that the model’s decisions are not arbitrary but are based on feature differences consistent with clinical epidemiology, providing internal support for the credibility of its warning signals within integrated surveillance systems.

### 4.7. Limitations

Several limitations pertain to the study design and data characteristics. First, the retrospective single-center design limits generalizability; the data originated from a single tertiary hospital in Beijing during a defined period, and external validation across institutions, regions, and time periods is needed. Radiology reporting practices, including terminology and style, may vary substantially across institutions, and model performance may degrade when applied to reports from different settings. Second, the PUE group comprised exclusively COVID-19 cases—a deliberate choice to simulate early outbreak detection—which introduces etiological homogeneity bias: the model may have learned features specific to SARS-CoV-2 rather than generalizable features of PUE arising from diverse etiologies. Future validation using PUE cases from multiple pathogens is essential. Third, the study lacks external validation; the test set was derived from the same institution and time period as the training data, potentially overestimating performance. The reported AUC of 0.986 (95% CI: 0.981–0.991), while encouraging, should be interpreted with caution; this high performance may partially reflect favorable data characteristics (e.g., consistent radiology reporting practices within a single institution and the relatively homogeneous PUE class composition) rather than inherent model generalizability. Fourth, the optimal decision threshold (0.08) was determined using the internal validation set, and the test set was used solely for final performance evaluation, following standard machine learning practice to avoid evaluation bias. Nevertheless, the threshold was optimized for this specific dataset and may not generalize to other settings without recalibration. Fifth, model performance depends on the quality and consistency of radiology text; variability in reporting language—including differences in the use of standardized lexicons, abbreviations, and descriptive styles—can affect feature extraction and model predictions.

Additional limitations concern the surveillance system design and the interpretation of results. First, the cluster alert rule (≥2 epidemiologically linked cases within 14 days) and the preliminary linkage algorithm are intentionally configured for high sensitivity, which may generate frequent false alerts in real-world deployment, and do not incorporate advanced statistical anomaly detection methods. Second, the training period overlapped with the COVID-19 pandemic, raising the possibility that the model captured pandemic-specific patterns. Third, the t-SNE and UMAP visualizations presented in Section 3.5 are intended for qualitative illustration only and should not be interpreted as quantitative evidence of model validity. Fourth, the conclusion that reviewing approximately 12% of reports (equivalent to 2.7 false positives per 100 reports) is manageable may not generalize across all healthcare settings; the acceptable workload for manual review depends on local resources, staffing levels, and existing surveillance infrastructure, and should be assessed on a site-specific basis. Despite these limitations, this study provides a proof-of-concept for NLP-based PUE surveillance and highlights key methodological considerations for future research.

### 4.8. Future Perspectives

Future research should focus on advancing this surveillance and predictive analytics system as a cornerstone of public health infrastructure. The primary direction is to conduct multi-center prospective implementation studies, evaluating the system’s integration feasibility, operational stability, warning accuracy, and actual effect on shortening public health response times in real hospital environments. Furthermore, to reduce false alerts while maintaining sensitivity, future iterations should incorporate more sophisticated cluster detection methods, such as spatial scan statistics (e.g., SaTScan) to identify statistically significant spatial clusters, time-series anomaly detection algorithms to establish dynamic baselines accounting for seasonal variations, and probabilistic record linkage to improve the accuracy of epidemiological associations. More importantly, exploring cross-institutional aggregation analysis of warning signals is essential. Signals from a single hospital may be weak, but the spatiotemporal aggregation of signals from multiple hospitals within a region can greatly enhance warning specificity and timeliness for epidemic detection. One promising approach is to build a regional, privacy-preserving collaborative data analysis platform. For example, a federated learning model developed in 2021 used vital signs, laboratory data, and chest X-rays to predict future oxygen needs in symptomatic patients with COVID-19 [43]. Federated learning (FL) enables training AI models on data from multiple sources while maintaining data anonymity, thereby removing many data-sharing barriers. Third, research is needed to establish a “warning–response” linkage mechanism. The model’s automatic warning signals should be directly pushable to the emergency command platforms of hospital infection control departments and local CDCs, triggering standardized preliminary investigation procedures, truly achieving a leap from “intelligent perception” to “intelligent response” in outbreak management.

## 5. Conclusions

For epidemic surveillance and early warning of emerging infectious diseases, a persistent global public health challenge remains. Traditional surveillance systems often exhibit a critical lag during the initial outbreak phase, delaying a timely response. Addressing this systemic limitation, the present study developed a real-time intelligent surveillance model based on deep learning, designed for the detection and early warning of emerging infectious diseases caused by unknown pathogens. Furthermore, an application scheme was devised for seamless, automated integration with hospital information systems and alignment with national surveillance frameworks. The optimal decision threshold, determined by the Matthews correlation coefficient (MCC) curve—a comprehensive performance metric—was established at 0.08. At this threshold, the model achieved a sensitivity of 89.8%, a specificity of 97.0%, and an area under the curve (AUC) of 0.986 (95% CI: 0.981–0.991). This performance represents an optimized balance between a high capture rate of high-risk cases and high signal reliability for outbreak prediction. A significant advancement of this research lies in translating a high-performance algorithmic model into an operational surveillance system: it functions as a backend service for real-time analysis of chest imaging text, and autonomously executes the cluster alert logic defined in national guidelines—specifically regarding “two or more epidemiologically linked cases of pneumonia of unknown etiology (PUE) occurring within 14 days.” This enables a fundamental shift from passive, delayed diagnostic reporting to proactive, real-time predictive surveillance. In summary, this study not only offers a novel methodological approach for early warning of emerging infectious diseases caused by unknown pathogens, but also delineates a clear translational pathway encompassing “technological innovation-system integration-alignment with regulatory frameworks.” Despite challenges for widespread implementation, such as the need for external validation and system adaptation across diverse healthcare settings, this research provides an innovative and significant solution for developing more automated, intelligent, and efficient surveillance and predictive analytics systems for emerging infectious disease Tepidemics.

## Figures and Tables

**Table 1:** Baseline table of research subjects.

	PUE (*n* = 980)	KEP (*n* = 7880)	*p*-Value
Demographic characteristic			
Age, mean (SD)	66.4 (22.5)	53.1 (19.7)	*p* < 0.001
Sex (%), female (%)	33.8%	32.0%	*p* = 0.232
Radiologic characteristic, *n* (%)			
Ground-glass opacity/high-density shadow, etc.	57.4%	35.5%	*p* < 0.001
Solid calcification/air bronchogram/fibrous cord shadow, etc.	21.8%	17.3%	*p* = 0.001
Tree-bud sign/millet nodules/large mass/cavity/pleural effusion	19.7%	20.4%	*p* = 0.633
Peripheral distribution/Subpleural distribution/Multiple lesions/Diffuse distribution/Lower lobe involvement	0.8%	0.4%	*p* = 0.047
Laboratory index			
Inflammation/Infection-related			
CRP (mg/L), mean (SD)	41.6 (57.1)	33.1 (45.8)	*p* < 0.001
IL-6 (pg/mL), mean (SD)	82.1 (181.5)	72.0 (165.6)	*p* = 0.001
WBC (×10^9^/L), mean (SD)	8.3 (5.4)	6.9 (7.4)	*p* < 0.001
NEU (×10^9^/L), mean (SD)	4.5 (4.0)	6.7 (5.0)	*p* < 0.001
LYM (×10^9^/L), mean (SD)	1.0 (0.7)	1.4 (1.2)	*p* < 0.001
NEU%, mean (SD)	40.2 (34.2)	74.0 (20.3)	*p* < 0.001
LYM%, mean (SD)	10.7 (14.8)	16.2 (13.8)	*p* < 0.001
MON%, mean (SD)	6.1 (3.9)	3.6 (4.5)	*p* < 0.001
MON (×10^9^/L), mean (SD)	0.5 (0.4)	0.5 (0.4)	*p* = 0.001
EOS%, mean (SD)	0.7 (1.3)	1.3 (2.6)	*p* < 0.001
EOS (×10^9^/L), mean (SD)	0.04 (0.08)	0.15 (0.59)	*p* < 0.001
BAS%, mean (SD)	0.3 (0.4)	0.3 (0.3)	*p* = 0.321
BAS (×10^9^/L), mean (SD)	0.02 (0.02)	0.03 (0.03)	*p* < 0.001
Red blood cell-related			
RBC (×10^12^/L), mean (SD)	4.1 (0.7)	3.7 (0.9)	*p* < 0.001
HGB (g/L), mean (SD)	125.3 (19.3)	115.0 (25.5)	*p* < 0.001
HCT (L/L), mean (SD)	0.36 (0.08)	0.31 (0.12)	*p* < 0.001
MCV (fL), mean (SD)	91.4 (7.2)	93.7 (8.0)	*p* < 0.001
MCH (pg), mean (SD)	30.6 (2.5)	31.2 (3.4)	*p* < 0.001
MCHC (g/L), mean (SD)	335.3 (13.4)	333.3 (19.7)	*p* = 0.001
RDW-CV (%), mean (SD)	13.2 (1.3)	15.1 (3.0)	*p* < 0.001
RDW-SD (fL), mean (SD)	41.3 (5.9)	50.7 (10.2)	*p* < 0.001
Platelet-related			
PLT (×10^9^/L), mean (SD)	189.4 (96.6)	149.5 (101.8)	*p* < 0.001
PDW (fL), mean (SD)	12.7 (2.5)	13.3 (3.2)	*p* < 0.001
MPV (fL), mean (SD)	10.7 (1.0)	10.7 (1.4)	*p* = 0.527
PCT (%), mean (SD)	0.20 (0.10)	0.16 (0.10)	*p* < 0.001
P-LCR (%), mean (SD)	30.2 (7.8)	31.7 (9.6)	*p* < 0.001

Note: All *p*-values are two-tailed. A *p*-value less than 0.05 indicates a statistically significant difference. Laboratory data have been verified for correct units and normalisation; the values reported are as obtained from the hospital’s laboratory information system. The wide standard deviation for NEU% in the PUE group reflects the heterogeneity of COVID-19 presentations, including a subset of patients with marked lymphocytosis relative to neutropenia, which is consistent with viral pneumonia patterns. All data were rechecked, and no transcription errors were identified.

**Table 2:** The performance of the model varies under different thresholds.

**Threshold**	**Accuracy**	**Sensitivity**	**Specificity**	**Precision**	**NPV**	**F1-Score**
0.01	0.915	0.98	0.907	0.568	0.997	0.719
0.02	0.941	0.964	0.938	0.661	0.995	0.784
0.03	0.948	0.939	0.949	0.694	0.992	0.798
0.04	0.953	0.929	0.956	0.722	0.991	0.813
0.05	0.955	0.923	0.959	0.736	0.99	0.819
0.06	0.957	0.908	0.963	0.751	0.988	0.822
0.07	0.961	0.898	0.969	0.782	0.987	0.836
0.08	0.962	0.898	0.97	0.789	0.987	0.84
0.09	0.962	0.888	0.971	0.795	0.986	0.839
0.1	0.962	0.872	0.973	0.799	0.984	0.834
0.12	0.962	0.862	0.974	0.805	0.983	0.833
0.14	0.962	0.862	0.975	0.809	0.983	0.835
0.16	0.962	0.847	0.976	0.814	0.981	0.83
0.18	0.961	0.837	0.977	0.816	0.98	0.826
0.2	0.962	0.832	0.978	0.827	0.979	0.83
0.25	0.964	0.821	0.982	0.852	0.978	0.836
0.3	0.962	0.786	0.984	0.86	0.974	0.821
0.35	0.962	0.781	0.985	0.864	0.973	0.82
0.4	0.961	0.77	0.985	0.863	0.972	0.814
0.45	0.96	0.755	0.985	0.865	0.97	0.807
0.5	0.96	0.75	0.987	0.875	0.969	0.808
0.55	0.959	0.74	0.987	0.873	0.968	0.801
0.6	0.959	0.73	0.988	0.883	0.967	0.799
0.7	0.959	0.724	0.989	0.887	0.967	0.798
0.8	0.958	0.704	0.99	0.896	0.964	0.789
0.9	0.954	0.668	0.99	0.891	0.96	0.764
1	0.889	0	1	0	0.889	0

Note: The thresholds shown in this table are for reference only to illustrate performance trade-offs. The optimal threshold (0.08) was selected based on the validation set. All performance metrics in this table were computed on the test set after threshold selection, using the fixed threshold of 0.08 for classification; values at other thresholds are shown solely for illustrative purposes and were not used for threshold optimization.

## Data Availability

Data is unavailable due to privacy and ethical restrictions.

## Abbreviations

The following abbreviations are used in this manuscript:

PUE	Pneumonia of Unknown Etiology
KEP	Pneumonia of Known Etiology
MCC	Matthews Correlation Coefficient
AUC	Area Under the (ROC) Curve
EID	Emerging Infectious Diseases

## References

[1] Hawryluck L., Gold W.L., Robinson S., Pogorski S., Galea S., Styra R. (2004). SARS control and psychological effects of quarantine, Toronto, Canada. Emerg. Infect. Dis..

[2] Wang W., Xu Y., Gao R., Lu R., Han K., Wu G., Tan W. (2020). Detection of SARS-CoV-2 in Different Types of Clinical Specimens. JAMA.

[3] Azhar E.I., Hui D.S., Memish Z.A., Drosten C., Zumla A. (2019). The middle east respiratory syndrome (MERS). Infect. Dis. Clin..

[4] Martin A., Markhvida M., Hallegatte S., Walsh B. (2020). Socio-economic impacts of COVID-19 on household consumption and poverty. Econ. Disasters Clim. Change.

[5] Thiam M.-M., Pontais I., Forgeot C., Pedrono G., Paget L.-M., Fouillet A., Caserio-Schönemann C. (2022). Syndromic surveillance: A key component of population health monitoring during the first wave of the COVID-19 outbreak in France, February–June 2020. PLoS ONE.

[6] Buehler J.W., Berkelman R.L., Hartley D.M., Peters C.J. (2001). Syndromic surveillance and bioterrorism-related epidemics. Emerg. Infect. Dis..

[7] Dai Y., Wang J. (2020). Identifying the outbreak signal of COVID-19 before the response of the traditional disease monitoring system. PLoS Negl. Trop. Dis..

[8] Dogra A.E.K., Munyasa W.L., Nguyen-Viet H., Grace D. (2023). Looking in all the wrong places: A rationale for signal detection for pandemics based on existing data sources. IJID One Health.

[9] Zhou C., Wang S., Wang C., Qiang N., Xiu L., Hu Q., Wu W., Zhang X., Han L., Feng X. (2025). Integrated surveillance and early warning system of emerging infectious diseases in China at community level: Current status, gaps and perspectives. Sci. One Health.

[10] Corrao G., Bonaugurio A.S., Bagarella G., Maistrello M., Leoni O., Cereda D., Gori A. (2025). Does syndromic surveillance assist public health practice in early detecting respiratory epidemics? Evidence from a wide Italian retrospective experience. J. Infect. Public Health.

[11] Proverbio D., Kemp F., Magni S., Goncalves J. (2022). Performance of early warning signals for disease re emergence: A case study on COVID-19 data. PLoS Comput. Biol..

[12] Yamanishi K., Xu L., Yuki R., Fukushima S., Lin C.H. (2021). Change sign detection with differential MDL change statistics and its applications to COVID-19 pandemic analysis. Sci. Rep..

[13] Varshney R.K. (2023). State of the Globe: Navigating the Impact of SARS-CoV-2 Mutations on COVID-19 Testing. J. Glob. Infect. Dis..

[14] Schlaberg R., Queen K., Simmon K., Tardif K., Stockmann C., Flygare S., Kennedy B., Voelkerding K., Bramley A., Zhang J. (2017). Viral Pathogen Detection by Metagenomics and Pan-Viral Group Polymerase Chain Reaction in Children With Pneumonia Lacking Identifiable Etiology. J. Infect. Dis..

[15] Villanueva-Miranda I., Xiao G., Xie Y. (2025). Artificial intelligence in early warning systems for infectious disease surveillance: A systematic review. Front. Public Health.

[16] Christaki E. (2015). New technologies in predicting, preventing and controlling emerging infectious diseases. Virulence.

[17] Markham S. (2025). Patient perspective on predictive models in healthcare: Translation into practice, ethical implications and limitations?. BMJ Health Care Inform..

[18] Peterson K.S., Dalton C., Kalvesmaki A., Vuong J., Gordon C., Nachimuthu S., Pugh M.J., Jones M.M. (2026). Identifying Early Signals From Emerging Public Health Events Using Natural Language Processing. Interdiscip. Perspect. Infect. Dis..

[19] Rani S., Kumar R., Panda B.S., Kumar R., Muften N.F., Abass M.A., Lozanović J. (2025). Machine Learning-Powered Smart Healthcare Systems in the Era of Big Data: Applications, Diagnostic Insights, Challenges, and Ethical Implications. Diagnostics.

[20] Fang Y., Zhang H., Xie J., Lin M., Ying L., Pang P., Ji W. (2020). Sensitivity of chest CT for COVID-19: Comparison to RT-PCR. Radiology.

[21] Yan L., Zhang H.T., Goncalves J., Xiao Y., Wang M., Guo Y., Sun C., Tang X., Jing L., Zhang M. (2020). An interpretable mortality prediction model for COVID-19 patients. Nat. Mach. Intell..

[22] Shen B., Yi X., Sun Y., Bi X., Du J., Zhang C., Quan S., Zhang F., Sun R., Qian L. (2020). Proteomic and metabolomic characterization of COVID-19 patient sera. Cell.

[23] Harmon S.A., Sanford T.H., Xu S., Turkbey E.B., Roth H., Xu Z., Yang D., Myronenko A., Anderson V., Amalou A. (2020). Artificial intelligence for the detection of COVID-19 pneumonia on chest CT using multinational datasets. Nat. Commun..

[24] Liang W., Yao J., Chen A., Lv Q., Zanin M., Liu J., Wong S., Li Y., Lu J., Liang H. (2020). Early triage of critically ill COVID-19 patients using deep learning. Nat. Commun..

[25] Chen J., Wu L., Zhang J., Zhang L., Gong D., Zhao Y., Chen Q., Huang S., Yang M., Yang X. (2020). Deep learning-based model for detecting 2019 novel coronavirus pneumonia on high-resolution computed tomography. Sci. Rep..

[26] Wang L., Lin Z.Q., Wong A. (2020). Covid-Net: A tailored deep convolutional neural network design for detection of covid-19 cases from chest x-ray images. Sci. Rep..

[27] Shapiro M., Landau R., Shay S., Kaminsky M., Verhovsky G. (2022). Early detection of COVID-19 outbreaks using textual analysis of electronic medical records. Int. J. Infect. Dis..

[28] McMurry A.J., Zipursky A.R., Geva A., Olson K.L., Jones J.R., Ignatov V., Miller T.A., Mandl K.D. (2024). Moving biosurveillance beyond coded data using AI for symptom detection from physician notes: Retrospective cohort study. J. Med. Internet Res..

[29] Hatachi T., Hashizume T., Taniguchi M., Inata Y., Aoki Y., Kawamura A., Takeuchi M. (2023). Machine Learning-Based Prediction of Hospital Admission Among Children in an Emergency Care Center. Pediatr. Emerg. Care.

[30] Kurita Y., Meguro S., Tsuyama N., Kosugi I., Enomoto Y., Kawasaki H., Uemura T., Kimura M., Iwashita T. (2023). Accurate deep learning model using semi-supervised learning and Noisy Student for cervical cancer screening in low magnification images. PLoS ONE.

[31] Chen H., Cheng Y.H., Yeh W.C., Chen Y.C., Tsai Y.W. (2025). A Machine Learning-Based Prognostication Model Enhances Prediction of Early Hepatic Encephalopathy in Patients With Noncancer-Related Cirrhosis: Multicenter Longitudinal Cohort Study in Taiwan. JMIR Med. Inform..

[32] Song K., Wu J., Xu M., Li M., Chen Y., Zhang Y., Chen H., Jiang C. (2025). An ensemble learning model to predict lymph node metastasis in early gastric cancer. Sci. Rep..

[33] Payman A.A., El-Sayed I., Rubio R.R. (2024). Exploring the Combination of Computer Vision and Surgical Neuroanatomy: A Workflow Involving Artificial Intelligence for the Identification of Skull Base Foramina. World Neurosurg..

[34] Deng J., Moskalyk M., Shammas-Toma M., Aoude A., Ghert M., Bhatnagar S., Bozzo A. (2024). Development of Machine Learning Models for Predicting the 1-Year Risk of Reoperation After Lower Limb On-cological Resection and Endoprosthetic Reconstruction Based on Data From the PARITY Trial. J. Surg. Oncol..

[35] Yang Z., Yang D., Dyer C., He X., Smola A., Hovy E. (2016). Hierarchical attention networks for document classification. Proceedings of the 2016 Conference of the North American Chapter of the Association for Computational Linguistics: Human Language Technologies.

[36] Awan J., Faherty L.J., Willis H.H. (2024). Navigating uncertainty in public health decisionmaking: The role of a value of information framework in threat agnostic biosurveillance. Health Secur..

[37] Rao S., Rashid A., Dafaalla M., Kandaswamy G., Kaddourah A. (2025). Refined selection of individuals for preventive cardiovascular disease treatment with a transformer-based risk model. Lancet Digit. Health.

[38] Phumkuea T., Wongsirichot T., Damkliang K., Navasakulpong A. (2023). Classifying COVID-19 Patients From Chest X-ray Images Using Hybrid Machine Learning Techniques: Development and Evaluation. JMIR Form. Res..

[39] Howroyd F., Veiga Sardeli A., Gao Smith F., Veenith T., Duggal N.A., Ahmed Z. (2025). Biomarkers for pneumonia after major trauma: A systematic review and meta-analysis. J. Intensive Care Soc..

[40] Phuong N.D., Tuyen N.T., Linh V.T.T., Nguyen N.N., Nguyen T.Q. (2025). Machine Learning Techniques in Chronic Kidney Diseases: A Comparative Study of Classification Model Performance. Bioinform. Biol. Insights.

[41] Arshad F., Schuemie M.J., Bu F., Minty E.P., Alshammari T.M., Lai L.Y.H., Duarte-Salles T., Fortin S., Nyberg F., Ryan P.B. (2023). Serially combining epidemiological designs does not improve overall signal detection in vaccine safety surveillance. Drug Saf..

[42] Antoñanzas J.M., Perramon A., López C., Boneta M., Aguilera C., Capdevila R., Gatell A., Serrano P., Poblet M., Canadell D. (2022). Symptom-Based Predictive Model of COVID-19 Disease in Children. Viruses.

[43] Dayan I., Roth H.R., Zhong A., Harouni A., Gentili A., Abidin A.Z., Liu A., Costa A.B., Wood B.J., Tsai C.-S. (2021). Federated learning for predicting clinical outcomes in patients with COVID-19. Nat. Med..

